# Effects of Production Systems and Seasons on Retail-Goat-Milk Fatty-Acid Composition and Nutritional Indices in Greece

**DOI:** 10.3390/ani12172204

**Published:** 2022-08-26

**Authors:** Eleni Kasapidou, Maria-Anastasia Karatzia, Paraskevi Mitlianga, Zoitsa Basdagianni

**Affiliations:** 1 Department of Agriculture, University of Western Macedonia, Terma Kontopoulou, 53100 Florina, Greece; 2 Research Institute of Animal Science, HAO–Demeter, 58100 Giannitsa, Greece; 3 Department of Chemical Engineering, University of Western Macedonia, Koila, 50100 Kozani, Greece; 4 Department of Animal Production, School of Agriculture, Aristotle University of Thessaloniki, 54124 Thessaloniki, Greece

**Keywords:** goat milk, retail, conventional, organic, chemical composition, fatty-acid composition, season, management, dairy

## Abstract

**Simple Summary:**

The consumption of goat milk is constantly increasing. However, there are not many studies on the nutritional profile of retail goat milk. This study examined the effects of production systems (conventional and organic) and seasons (spring, summer, autumn and winter) on the fatty acid composition and nutritional indices related to the healthy fat consumption of fluid goat milk sold in the supermarkets. The production system did not significantly affect milk fatty-acid composition nor its nutritional value, whereas seasonal effects were observed. Overall, retail goat milk had a desirable nutritional value, irrespective of production system and season.

**Abstract:**

An increased consumer interest in goat milk has been registered in recent years due to its health-related benefits. This study aimed to investigate the effects of production systems and seasons on fatty-acid composition and nutritional indices for the healthy fat consumption of retail goat milk. The study lasted one year, and milk samples (*n* = 160) from 14 brands (10 conventional and 4 organic) were collected on a monthly basis. Organic milk had a marginally lower fat and protein content compared with conventionally produced milk according to the declared chemical composition. The production system did not significantly affect milk fatty-acid composition and its nutritional value, indicating that there were no major differences in the feeding strategies between conventionally and organically raised ewes. Seasonal effects were observed in the fatty-acid profile and the nutritional indices. Lower content of saturated fatty acids (SFAs), hypocholesterolemic/hypercholesterolemic ratio (h/H) and linoleic acid/α-linolenic acid (LA/ALA) ratio values were observed in summer. Retail goat milk had a desirable nutritional value, irrespective of production system and season, due to the following characteristics: low atherogenic index (AI) and LA/ALA ratio values, and high h/H ratio, desirable fatty acid (DFA) values and health-promoting index values. In conclusion, neither production nor season significantly impacted the fatty-acid composition and the fat-consumption nutritional indices of retail goat milk produced in Greece.

## 1. Introduction

Dairy goat farming is rapidly increasing worldwide due to the growing demand for goat dairy products [1]. Following this trend, goat-milk production has also increased by almost 20% in the period of 2016–2020 in Greece. Furthermore, the increase in organic milk production is impressive, reaching 164% in the same period (Table 1; Hellenic Agricultural Organization—Demeter; personal communication). Consumers prefer goat-milk products due to their health and nutritional benefits, such as greater digestibility, enhanced lipid metabolism and lower allergenicity [2,3]. Regarding fatty-acid composition, goat milk has higher contents of monounsaturated, polyunsaturated and medium-chain fatty acids beneficial for human health than cow milk [4,5]. Furthermore, goat milk has a lower cholesterol content than other types of milk [6].

The effects of factors such as breed, diet, farming system and season on milk composition was documented in the review studies by Park [7], Goetsch et al. [8], and Clark and García [9]. However, the published data on goat-milk composition refer to samples from farm-based studies. Moreover, Butler et al. [10] stated that it is unclear whether the results of such studies can be extrapolated to evaluate the milk quality available to consumers, because (a) individual farms chosen for sampling may not be representative of the production system within a country and (b) processing conditions within the supply chain may influence milk composition.

Information on goat-milk quality at the retail level is limited. Previous work by Stergiadis et al. [11] showed a seasonal variation in the nutritional value of retail goat milk in the United Kingdom. Pittau et al. [12] studied the fatty-acid composition of retail goat milk in Italy and reported differences between small-scale milk producers and industrial plants. Regarding Greece, there are no studies on the nutritional value of retail fluid goat milk, although conventional or organic milk is widely available in food stores. Therefore, the study aimed to investigate the effects of production systems (conventional and organic), seasons (spring, summer, autumn and winter) and their interaction on the fatty-acid composition of retail goat milk produced in Greece. A secondary objective was to evaluate the nutritional value of milk in relation to current guidelines for healthy fat consumption.

## 2. Materials and Methods

### 2.1. Sampling

Milk samples (*n* = 160) from 14 brands were collected every month from May 2019 to April 2020 from four major supermarket retailers located in Northern Greece. All available goat-milk samples that were sold in supermarket retailers were included in the study. Samples were either whole or reduced fat homogenised milk, and were either conventionally (*n* = 10 brands) or organically produced (*n* = 4 brands). Milk samples were either pasteurized, had extended shelf life or were ultra-high temperature (UHT) processed. Two brands of conventionally produced milk were private (retail) labels. Furthermore, selected samples had to meet the following criteria: (a) to be widely available in food stores and (b) to bear the “Greek Produce” mark, which certifies the origin of good and services produced in Greece. The “Greek Produce“ mark is an official trademark of the Greek State and is awarded on the basis of the award regulations, separately for each category of goods and services. Food stores (supermarkets) were located within a 5 km radius from the laboratory where samples were stored and analyzed. Following purchase, milk samples were placed in isothermal containers with ice packs and transported to the laboratory within 1 h. On arrival at the laboratory, milk samples were thoroughly mixed and decanted into three 15 mL Falcon tubes that were stored at −20 °C prior to analysis. The declared milk composition and the price of each milk sample were noted. Care was taken in purchasing samples from the same brand from the same retailer each month to eliminate pricing discrepancies across retailers. The listed prices were regular prices and did not include any discounts or promotions. The characteristics of the milk samples are presented in Table 2. Milk from certain brands was not available in winter months, and this was attributed to the fact that milk was used for kid feeding following lambing.

### 2.2. Determination of Fatty-Acid Composition

Milk samples were thawed overnight at 4 °C, and the following day, milk lipids were extracted with a chloroform/methanol solution (1:2 *v*/*v*) according to the method by Bligh and Dyer [13]. The solvents contained 0.01% (*wt*/*v*) t-butyl-hydroxytoluene (BHT) to prevent the oxidation of unsaturated fatty acids during extraction. Fatty-acid methyl esters were prepared from the extracted lipids via the base-catalyzed methanolysis of the glycerides using KOH in methanol, according to the ISO–IDF 15884 method [14] of the International Organization for Standardization. Fatty-acid methyl ester analyses were performed with Agilent Technologies 6890N GC (Agilent Technologies, Inc., Santa Clara, CA, USA) equipped with a flame ionization detector (FID) and a 60 m × 0.25 mm i.d., 0.25 μm film thickness DB-23 (50% Cyanopropyl 50% dimethyl polysiloxane) capillary column (model number: Agilent 122 2362). The injector temperature was set at 250 °C. The oven temperature was programmed to increase from 110 °C (held for 6 min) to 165 °C at 1 °C/min (held for 13 min), to 195 °C at 15 °C/min (held for 22 min) and to 230 °C at 7 °C/min (held for 7 min). The carrier gas was helium at 0.7 mL/min, and the injection volume was set at 3 μL; the split ratio was 1:50. The injection was performed using an Agilent 7683 Series auto-sampler. Fatty acids were identified using three commercial standard mixtures: (a) 37 component FAME mix (Supelco; 47885-U), (b) PUFA-2, Animal source (Supelco; 47015-U) and (c) a mixture of *cis*- and *trans*-9,11- and -10,12-octadecadienoic acid methyl esters (Sigma; O5632–250MG) (Sigma-Aldrich, Taufkirchen, Germany) as reference standards. Fatty acids were quantified using peak-area measurement, and the results were expressed as percent (%) of the total peak areas for all identified acids.

Saturated fatty acids were also classified into short-, medium- and long-chain fatty acids as follows:

- Short-chain saturated fatty acids (SCSFAs) = C6:0 + C8:0 + C10:0 + C11:0;
- Medium-chain saturated fatty acids (MCSFAs) = C12:0 + C13:0 + C14:0 + C15:0 + C16:0;
- Long-chain saturated fatty acids (MCSFAs) = C17:0 + C18:0 + C20:0 + C22:0 + C23:0 + C24:0.

### 2.3. Nutritional Indices

The fatty-acid profile was used to calculate the nutritional indices related to healthy fat consumption. The applied indices were those reported in the recent review by Chen and Liu [15] for ruminant dairy products. Chen and Liu [15] compiled research on fatty-acid profiles, which was published since 2000, for a better comprehension of the implications and applications of various nutritional indices.

Atherogenicity index:

AI = [C12:0 + (4 × C14:0) + C16:0]/ΣUFA(1)

Thrombogenicity index:

TI = (C14:0 + C16:0 + C18:0)/[(0.5 × ΣMUFA) + (0.5 × Σ*n*−6PUFA) + (3 × Σ*n*−3PUFA) + (*n*−3/*n*−6)] (2)

Hypocholesterolemic:hypercholesterolemic fatty-acid ratio (h/H):

h/H = (C18:1*n*−9 *cis* + ΣPUFA)/(C12:0 + C14:0 + C16:0) (3)

Health-promoting index:

HPI = ΣUFA/[C12:0 + (4 × C14:0) + C16:0](4)

Linoleic acid/α-linolenic acid ratio:

LA/ALA = C18:2 *n*−6/C18:3 *n*−3 (5)

Trans fatty acids:

TFA = ΣTFA(6)

Polyunsaturated fatty acid/saturated fatty acid ratio:

PUFA/SFA = ΣPUFA/ΣSFA(7)

Finally, the desirable fatty acid (DFA) index was calculated according to the formula by Rhee [16] as follows:

DFA = ΣUFA + C18:0(8)

### 2.4. Statistical Analysis

General linear models were used to investigate the differences in milk fatty-acid composition and lipid quality nutritional indices due to (a) the production system and (b) the season. Production system and season were fixed factors. The effect of the interaction between the main factors was also assessed. The effect of the season was compared using Tukey’s test. The results were considered to be significant when the *p*-values were <0.05. The results are presented as least square means. SPSS software (version 26.0; SPSS Inc., Chicago, IL, USA) was used for data analysis. The distribution of the samples was mapped in a two-dimensional space using a Principal Component Analysis (PCA) in order to see if there was a separation among different groups. This analysis was performed using R software (version 4.2.1; R Foundation for Statistical Computing, Vienna, Austria) and factoextra (version 1.0.7) [17].

## 3. Results and Discussion

### 3.1. Milk Characteristics

The characteristics of the milk samples in relation to the declared energy (kj/100 mL) and the composition of the milk (g/100 mL), and the average price per production system are presented in Table 3. In this study, 71.43% of the samples were produced with conventional production systems and the remaining 28.57% with organic production systems. The milk nutritional value in relation to energy and chemical composition was similar between the two production systems. Average fat and protein contents were slightly lower in organically produced milk, whereas sugar content was slightly higher than the conventionally produced one. Fat content ranged from 2.8 to 4.1 g/100 mL for both production systems, while protein content ranged from 3.6 to 4.6 g/100 mL in conventionally produced milk and from 3.5 to 4.1 g/100 mL in organically produced milk. Carbohydrate content ranged from 4.1 to 4.6 g/100 mL in milk obtained with both production systems, while sugar content varied from 3.6 to 4.5 g/100 mL in conventional milk and from 3.8 to 4.5 g in organic milk. In the presented study, the chemical composition was expressed in g/100 mL, in accordance with European Union Regulation No. 1169/2011 [18] for food labeling. However, in order to compare the findings of this study with other studies/reports, the FAO/INFOODS guidelines were applied to convert the composition from g/100 mL to g/100 g (Table S1, Supplementary Material). In detail, goat whole-milk density (1.08 g/mL) was used (FAO/INFOODS Density Database; version 2.0) [19]. Protein and lactose contents were similar to those reported by Hellenic Agricultural Organization—Demeter [20] for raw goat milk for years 2019 and 2020 (protein, 3.66 g/100 g, and lactose, 4.50 g/100 g, for year 2019; protein, 3.68 g/100 g, and lactose, 4.46 g/100 g, for year 2020). According to the United States Food Composition Tables [21], whole-milk composition was as follows: protein, 3.56 g/100 g, and carbohydrates and sugars, 4.45 g/100 g. However, Stergiadis et al. [11] reported significantly lower protein content (27%, that is, 2.85 g/100 g) in conventional retail goat milk in the United Kingdom and attributed the lower protein content to genetic differences between goats in different countries. With regard to fat content, no comparisons could be made, since retail milk is sold with a standardized fat content. However, fat content in the study by Stergiadis et al. (2019) [11] was 3.58 g/100 g and similar to the fat content of conventional milk in this study. The fat content reported in the USDA Food Composition Tables [21] was 4.14 g/100 g. Finally, according to Hellenic Agricultural Organization—Demeter [20], the fat contents of raw unprocessed milk were 4.82 and 4.80 g/100 g in years 2019 and 2020, respectively.

The average price was 2.41 EUR/lt. Organic milk was 24.4% more expensive than conventional milk (2.25 vs. 2.80 EUR/lt). However, 20% of the conventionally produced milk samples were private-labeled. When milk price was compared among the branded samples of both production systems, the average price per liter of milk rose to 2.39 EUR/lt for conventionally produced milk, and subsequently, organic milk was 17.15% more expensive. It was reported that although the price of goat milk is generally higher than that of cow milk, consumers buying goat milk either as a health-promoting or a specialty food are not influenced by product price [22,23].

### 3.2. Effects of Production Systems and Seasons on Milk Fatty-Acid Composition

Milk fatty-acid composition as individual fatty acids and as different lipid classes is presented in Table 4 and Table 5, respectively. Capric acid (C10:0), myristic acid (C14:0), palmitic acid (C16:0) and stearic acid (C18:0) were the predominant saturated fatty acids in both production systems and in all seasons. Oleic acid (C18:1 *cis*-9) was the major monounsaturated fatty acid, whereas linoleic acid (C18:2 *n*−6 *cis*) was also the predominant polyunsaturated fatty acid in both production systems and in all seasons. The production system affected (*p* < 0.01–*p* < 0.05) the proportions of capric acid (C10:0), lauric acid (C12:0), myristic acid (C14:0), heptadecenoic acid (C17:1), stearic acid (C18:0), vaccenic acid (C18:1 *trans*-11) and arachidic acid (C20:0). Significant differences (*p* < 0.001–*p* < 0.05) were also observed in the percentages of caproic acid (C6:0), caprylic acid (C8:0), capric acid (C10:0), myristic acid (C14:0), pentadecanoic acid (C15:0), pentadecenoic acid (C15:1), palmitic acid (C16:0), palmitoleic acid (C16:1), heptadecanoic (C17:0), heptadecenoic acid (C17:1), vaccenic acid (C18:1 *trans*-11), oleic acid (C18:1 *cis*-9), linolelaidic acid (C18:2 *n*−6 *trans*), linoleic acid (C18:2 *n*−6 *cis*) and α-linolenic acid (C18:3 *n*−3) due to the effect of the season. The effect of the season on fatty-acid composition was more profound in relation to the effect of the production system. Finally, the interactions between production systems and seasons affected only the levels of vaccenic acid (C18:1 *trans*-11) and oleic acid (C18:1 *cis*-9).

Neither the production system nor the season affected (*p* ≥ 0.05) the levels of conjugated linoleic acid (CLA) (C18:2 *cis*-9 *trans*-11). There are contradictory findings on the effect of the production system on goat-milk-conjugated linoleic acid (CLA) (C18:2 *cis*-9 *trans*-11) levels in the literature. In detail, Tudisco et al. [24] and Tzamaloukas et al. [25] reported significantly higher levels of conjugated linoleic acid (CLA) (C18:2 *cis*-9 *trans*-11) in organically produced milk, whereas Tsiplakou et al. [26] reported no production-system effects in a trial where conventionally and organically produced milk represented the feeding systems applied in Greece. There were also no seasonal effects on the levels of conjugated linoleic acid (CLA) (C18:2 *cis*-9 *trans*-11). Our findings are in agreement with those obtained by Tsiplakou et al. [26,27] and Tzamaloukas et al. [25], who reported no seasonal variations in the percentage of conjugated linoleic acid (CLA) (C18:2 *cis*-9 *trans*-11). Although pasture feeding can increase conjugated linoleic acid (CLA) (C18:2 *cis*-9 *trans*-11) concentration in milk fat [28], parameters such as the type of forage, variations in pasture availability and the stage of grass growth maturity could affect the resulting CLA concentration in milk fat [27,29] and explain the fact that there were neither production-system nor seasonal effects. Furthermore, the fact that neither the production system nor the season affected the levels of conjugated linoleic acid (CLA) (C18:2 *cis*-9 *trans*-11) can be also related to the lack of production-system and seasonal effects on the Δ^9^-desaturase activity (Stearoyl-CoA desaturase) C14:1/C14:0. Δ^9^-desaturase-activity C14:1/C14:0 is considered the best indicator, since myristic acid (C14:0) in milk is produced via de novo synthesis in the mammary gland [30]. The majority of conjugated linoleic acid (CLA) (C18:2 *cis*-9 *trans*-11) is endogenously synthesized from vaccenic acid (C18:1 *trans*-11) by the Δ^9^-desaturase enzyme [26].

With regard to Δ^9^-desaturase activity, the highest ratio was observed for C18:1/C18:0. The same patterns of the Δ^9^-desaturase-activity ratios of C14:1/C14:0, C16:1/C16:0, C18:1/C18:0 and conjugated linoleic acid (CLA)/vaccenic acid were also reported by Tsiplakou et al. [26] and Stergiadis et al. [11]. The production system had no effects (*p* ≥ 0.05) on Δ^9^-desaturase, but significant seasonal effects (*p* < 0.001–*p* < 0.05) on C16:1/C16:0, C18:1 *cis*-9/C18:0 and CLA/VA activities were observed. Tudisco et al. [31] also reported that the production system did not affect Δ^9^-desaturase activity, whereas Tsiplakou et al. [26] found differences in the Δ^9^-desaturase-activity ratio of C18:1/C18:0 between conventionally and organically produced milk. There was a significant seasonal effect (*p* < 0.001; *p* < 0.05) on Δ^9^-desaturase. In the present study, the highest Δ^9^-desaturase-activity values were observed in autumn, and this is in apparent disagreement with the current knowledge that there is an increase in Δ^9^-desaturase activity in animals fed fresh grass, i.e., cows in spring and summer [30]. In general, the factors affecting Δ^9^-desaturase activity in goats are not well understood, and it is important to note that milk fatty-acid secretion and milk fat-lipolysis responses to physiological and nutritional factors differ markedly between cows and goats [32]. The interaction of production systems and seasons did not influence (*p* ≥ 0.05) Δ^9^-desaturase activity.

In relation to the effects of production systems and seasons on milk lipid classes, examination of the individual lipid classes of saturated fatty acids showed that the production system affected (*p* < 0.05) the levels of long-chain saturated fatty acids (LCSFAs). In the present study, lower levels of LCSFAs were found in organically produced milk. The effect of the production system on the subclasses of saturated fatty acids reported in the literature is not consistent. Tsiplakou et al. [26] reported significantly lower levels of SCSFAs and MCSFAs and higher levels of LCSFAs in organic milk than in conventionally produced milk, whereas Tzamaloukas et al. [25] reported significantly lower levels of SCSFAs, MCSFAs and LCSFAs in organic milk. Additionally, a seasonal effect (*p* < 0.05) was observed in the levels of MCSFAs.

The production system did not affect lipid classes such as saturated fatty acids (SFAs), monounsaturated fatty acids (MUFAs), polyunsaturated fatty acids (PUFAs), odd-chain fatty acids (OCFAs), unsaturated fatty acids (UFAs), and *n*−3 and *n*−6 fatty acids. Pittau et al. [12] and Stergiadis et al. [11] reported similar levels for individual lipid classes in retail goat milk. In the study by Pittau et al. [12], the average composition was 68.4% SFAs, 21.3% MUFAs and 5.3% PUFAs, whereas according to Stergiadis et al. [11], milk fat consisted of 70.3% SFAs, 25.3% MUFAs and 4.4% PUFAs. Kučević et al. [33] reported that organically produced milk had a significantly higher concentration of SFAs and a lower concentration of UFAs, and Tsiplakou et al. [26] reported higher but not significantly different levels of MUFAs and PUFAs in organically produced milk. Lopez et al. [34] found a significantly higher SFA level and significantly lower MUFA and PUFA levels in low-input organically produced milk than in low-input conventionally produced milk.

A seasonal effect (*p* < 0.05) on UFAs was observed, and a highly significant effect (*p* < 0.01) on *n*−6 fatty acids was observed. The highest level of *n*−6 fatty acids was observed in winter. This finding is in contrast with the results obtained by Milewski et al. [35], who reported a higher level of *n*−6 fatty acids in summer milk from alpine goats and associated this difference with grazing.

The combination of production systems and seasons did not have an impact (*p* ≥ 0.05) on the lipid classes.

The Principal Component Analysis showed that there were no strong common patterns among individual fatty acids, production systems and seasons in goat milk (Supplementary Material, Figures S1–S3).

### 3.3. Effects of Production Systems and Seasons on Milk Fat Nutritional Indices

The effects of the production system and the season on the lipid quality of milk are presented in Table 6. There were no differences (*p* ≥ 0.05) either due to the production system or due to the season in both the atherogenicity (AI) and thombogenicity (TI) indices. In a similar study, Tzamaloukas et al. [25] reported that both the production system and the season significantly affected the AI of goat milk, with a higher AI being observed in conventionally produced milk and in milk produced in autumn. In retail milk samples, Stergiadis et al. [11] reported a higher AI (2.56) for conventionally produced milk. Additionally, Pittau et al. [12] reported AI values in the range of 2.27–2.91 for marketed goat milk in Sardinia. Finally, Cossignani et al. [36] reported AI values ranging from 2.6 to 3.2 for goat-milk samples from the Umbrian market. Pietrzak-Fiećko and Kamelska-Sadowska [37] also reported a higher AI value (3.17) for milk samples collected from small individual farms in Poland. Finally, Tsiplakou et al. [26] reported a significantly lower AI for organic goat milk than for conventional goat milk produced in winter. The TI was similar (3.13) to the value also reported by Stergiadis et al. [11] and within the range of 2.70–3.20 reported by Pittau et al. [12] for retail milk.

The AI depicts the relationship between saturated fatty acids (SFAs), such as lauric (C12:0), myristic (C14:0) and palmitic acids (C16:0), which are considered pro-atherogenic, and unsaturated fatty acids (UFAs), which are considered anti-atherogenic because they inhibit plaque formation and lower the levels of phospholipids, cholesterol and esterified fatty acids. The TI refers to fatty acids’ thrombogenic potential, indicating their tendency to form clots in blood vessels [38]. Low values, and preferably <3, of both indices are beneficial for human health [39]. In the present study, the TI value marginally exceeded the recommended value.

With regard to the hypocholesterolemic: hypercholesterolemic (h/H) ratio, a significant (*p* < 0.05) production-system effect and a highly significant (*p* < 0.001) seasonal effect were observed. A higher h/H ratio was found in conventionally produced milk than in organically produced milk. Lower h/H ratio values were observed in milk produced in autumn, winter and spring than in milk produced in summer. The mean h/H ratio for the production system was 0.63, whereas the mean h/H ratio for the season was 0.62. Pietrzak-Fiećko and Kamelska-Sadowska [37] reported an h/H ratio of 0.59 for goat milk. Since the h/H ratio describes the relationship between the hypocholesterolemic and the hypercholesterolemic fatty acids, high values are desirable. Highly significant effects (*p* < 0.001) of the interaction between production systems and seasons were found for the h/H ratio.

Neither the production system nor the season had a significant impact (*p* ≥ 0.05) on the health-promoting index (HPI). The HPI, irrespectively of the production system and the season, was within the range (0.34–0.45) reported by Claps et al. [40] for goats fed either on pasture or on pasture supplemented with concentrates. In the latter study, higher HPI values were found in spring and summer and for pasture-fed animals. Dairy products having a high HPI value are considered to be healthier for humans, since the HPI is the reverse of the TI [15].

The linoleic acid/α-linolenic acid (LA/ALA) ratio was not affected (*p* ≥ 0.05) by the production system, whereas it was highly significantly affected (*p* < 0.001) by the season. Significantly higher LA/ALA ratio values were observed in autumn and winter than in spring and summer. Średnicka-Tober et al. [41] reported that high intakes of concentrates, which are associated with winter diets [28], increase the level of linoleic acid, thus leading to a higher LA/ALA ratio. Tzamaloukas et al. [25] reported a tendency for a seasonal effect in the LA/ALA ratio in bulk-tank goat milk collected from conventional and organic farms. The LA/ALA ratio values were within the reported range (3.81–7.44) for goat milk produced from ewes fed concentrates [42,43] but remarkably lower than the values (10.37–11.37) reported by Tzamaloukas et al. [25] for goat milk either conventionally or organically produced throughout the year. Lower LA/ALA ratio values are preferable because the LA/ALA ratio is related to higher levels of *n*−3 fatty acids that are beneficial for health, such as α-linolenic acid (C18:3 *n*−3), which is the predominant acid of the *n*−3 lipid class in goat milk. Furthermore, a high LA/ALA ratio raises the risk of a variety of other chronic disorders, including cancer, inflammatory and autoimmune diseases, and cardiovascular diseases [41].

There were no significant differences (*p* ≥ 0.05) either due to the production system or due to the season in the trans fatty acid (TFA) index. Intake of trans fatty acids is associated with an increased risk of coronary heart disease, and according to the EFSA [44], the intake of TFAs should be sufficiently reduced within a nutritionally adequate diet. Trans fatty acids are naturally present in goat milk; thus, the above-reported recommendation applies to the intake of industrially produced TFAs. Pittau et al. [12] and Stergiadis et al. [11] reported a significantly higher level of TFAs in retail goat milk (3.6 and 2.46 g/100 g of fat, respectively).

Higher levels (*p* < 0.05) of DFAs were found in conventionally produced milk than in organically produced milk. However, no seasonal effects (*p* ≥ 0.05) were observed. According to Osmari et al. [45], higher levels of unsaturated fatty acids in the diet can increase the DFA concentration in milk. DFAs represent the sum of stearic acid (C18:0) and unsaturated fatty acids, which are all anti-atherogenic because they lower plasma cholesterol and triacylglycerols [15,46].

There were no effects of neither the production system nor the season on the PUFA/SFA ratio, which had unfavorably low values and was far below the Department of Health’s recommendation of 0.45 [47]. The PUFA/SFA ratio is most commonly used to assess the nutritional value of ingested fat. Foods with a PUFA/SFA ratio higher than 0.45 are recommended to prevent cardiovascular and other chronic diseases. In general, ruminant milk has a low PUFA/SFA ratio, and according to Gibson et al. [48], who reviewed cohort studies, there is no consistent evidence that consumption of dairy products is associated with a higher risk of cardiovascular disease.

In the present study, the production system did not affect milk fatty-acid composition and milk fat nutritional indices overall. In detail, organic farming did not result in milk having a lower concentration of SFAs or a higher concentration of UFAs, as it was reported in other studies for ruminant milk [24,26]. Additionally, seasons affected the milk fatty-acid profile and the nutritional value but not in the expected manner, i.e., improved nutritional indices for milk produced in spring and summer, due to grazing, in comparison with milk produced in winter [10,25]. The findings of this study showed that there were no significant differences in the feeding strategies between conventional and organic dairy farms. Goats graze green forages, naturally grown pastures and shrubs from spring to autumn, and supplementary concentrate feed is provided when required. In the winter months, when the available fresh forage is limited, animals are fed concentrates and conserved forages (silage). Usually, concentrates are either commercially produced, home-produced or a combination of both.

Another parameter that should be taken into account is the management system of the farm, i.e., high and low input within the same production system. Lopez et al. [34] reported differences in the fatty-acid composition in milk produced in high- and low-input conventional farms. The latter workers found that milk from low-input production systems, either organic or conventional, was richer in favorable nutritional components than milk from a high-input system. The inconsistent results between the different studies on the effects of the production system and the season on milk fatty-acid composition may also be due to the high variety of feeds used and, particularly in the case of organic milk, to the fact that producers aim to achieve the organic standards at a reasonable cost. According to European Union legislation [49], livestock should have permanent access to pasture whenever the conditions allow for it to happen or should have permanent access to roughage. Furthermore, at least 60% of the dry matter in the daily rations of herbivores should consist of roughage, fresh or dried fodder, or silage. The forage-to-concentrate ratio in the daily feed, the type of flora in the grazing site and the type of grass (fresh or silage) also affect the milk fatty-acid composition in ruminants. A higher forage-to-concentrate ratio results in improved fatty-acid composition [34]. Kusche et al. [50] reported improved fatty-acid composition in conventionally and organically produced cow milk from low-input farms and associated that to a better forage-to-concentrate ratio as well as the use of fresh forage instead of the conserved forages that are usually offered in high-input farms. Moreover, according to Barłowska et al. [51], goats grazing on natural mountain pastures with increased floristic diversity, and in particular the presence of more species of meadow plants and herbs, produce milk with improved nutritional content. Furthermore, fresh grass is generally less mature, containing higher levels of PUFAs in comparison to grass used for silage making [52], thus affecting the biohydrogenation pathways [53].

## 4. Conclusions

The present work is the first large-scale study examining the effects of production systems and seasons on goat milk fatty acid composition produced in Greece. Milk samples were either conventionally or organically produced as declared on the product label, and no background information on dairy management was available. The production system did not significantly affect milk fatty-acid composition nor the nutritional indices regarding healthy fat consumption. The effect of the season was also limited, and the beneficial effect of grazing, associated with milk produced in the spring and summer months, was not observed. Finally, there were no interactions between the production system and the season in terms of milk fatty-acid composition and nutritional indices overall. The results indicated that there were no major differences in the feeding strategies between conventionally and organically raised ewes throughout the year. Goat milk has a desirable nutritional value according to the nutritional indices. According to the results of the present study, the nutritional profile of goat milk produced in Greece can be further improved with changes in the animal diet such as supplementation with oil seeds, inclusion of silage, etc. Future research on parameters such as vitamin contents, mineral contents, bioactive compounds and sensory characteristics is required in order to differentiate the effects of production systems and seasons on fluid goat milk. Additionally, the identification of potential novel value chains and the upcycling of agricultural by-products as feed ingredients, along with the highlighting of local goat production systems and indigenous breeds, may provide fluid milk and dairy products with wider recognition, acceptance and added value for consumers, thus aiding the sustainability of the sector.

## Figures and Tables

**Table 1:** Goat-milk production (1000 kg) according to the production system (years 2016–2020) (Hellenic Agricultural Organization—Demeter, 2021; personal communication).

Year	Production System		Total
	Conventional	Organic	
2016	128,962	2754	131,715
2017	136,941	3401	140,342
2018	148,549	4787	153,336
2019	138,637	5733	144,370
2020	150,291	7274	157,565

**Table 2:** Characteristics of milk samples.

Brand No.	Production System	Shelf Life	Label
1	Organic	Extended shelf life	Branded
2	Conventional	Extended shelf life	Branded
3	Organic	7 days	Branded
4	Conventional	7 days	Branded
5	Organic	7 days	Branded
6	Conventional	7 days	Branded
7 ^1^	Conventional	7 days	Branded
8	Conventional	10 days	Private
9	Conventional	Extended shelf life	Branded
10 ^1^	Conventional	7 days	Branded
11	Conventional	Long life	Branded
12 ^2^	Conventional	7 days	Branded
13	Conventional	7 days	Private
14 ^3^	Organic	7 days	Branded

^1^ Νο samples available in November and December. ^2^ Νο samples available in December. ^3^ Brand was included in the study from August onwards.

**Table 3:** Declared energy (kj/100 mL), chemical composition (g/100 mL) and price (EUR/lt) of retail goat milk purchased in Greece in 2019–2020 for 12 months.

Brand No.	Production System	Energy (kj/100 mL)	Chemical Composition (g/100 mL)					Price (EUR/lt)
			Fats	SFAs ^1^	Proteins	Carbohydrates	Sugars	
1	Organic	269	3.5	2.2	3.5	4.6	4.5	2.68
2	Conventional	269	3.5	2.4	3.6	4.6	4.5	2.38
3	Organic	286	4.1	3.2	3.8	4.1	4.1	2.83
4	Conventional	286	4.1	3.2	3.8	4.1	4.1	2.49
5	Organic	279	2.8	2.2	3.8	4.4	4.4	2.89
6	Conventional	279	2.8	2.2	3.8	4.4	4.4	2.67
7	Conventional ^2^	267	3.5	2.2	3.8	4.5	4.5	2.30
8	Conventional	284	4.0	2.9	3.7	4.3	4.3	1.76
9	Conventional	269	3.5	2.3	3.7	4.5	4.5	2.30
10	Conventional	274	3.7	2.6	3.6	4.5	4.5	2.40
11	Conventional	266	3.5	2.15	4.4	4.4	3.6	2.41
12	Conventional	267	3.5	2.2	4.5	4.5	3.6	2.19
13	Conventional	267	3.5	2.2	4.5	4.5	3.6	1.62
14	Organic ^2^	275	3.5	2.2	4.1	4.1	3.8	2.81
Average (conventional)		272.80	3.56	2.44	3.94	4.43	4.16	2.25
Average (organic)		277.25	3.48	2.45	3.80	4.30	4.20	2.80
Average (all samples)		274.07	3.54	2.44	3.90	4.39	4.17	2.41

^1^ SFA, saturated fatty acids. ^2^ Compositional data are provided in g/100 g of milk.

**Table 4:** Effects of production system and season on milk fatty-acid composition (% of total identified fatty acids) and *p*- values for the main effects and their interaction.

Fatty Acid	Production Systems		Seasons				Main Effects		Interaction
	Conventional *n* = 94	Organic *n* = 66	Spring *n* = 41	Summer *n* = 40	Autumn *n* = 42	Winter *n* = 37	PS	S	PS × S
C6:0	1.608	1.589	1.642 ^a^	1.651 ^a^	1.589 ^a,b^	1.512 ^b^	NS	*	NS
C8:0	2.227	2.286	2.344 ^a^	2.257 ^a,b^	2.182 ^b^	2.243 ^a,b^	NS	*	NS
C10:0	8.534	8.902	9.002 ^a^	8.712 ^a,b^	8.447 ^b^	8.711 ^a,b^	**	*	NS
C11:0	0.199	0.196	0.193	0.205	0.198	0.192	NS	NS	NS
C12:0	4.088	4.370	4.209 ^a^	4.080 ^a,b^	4.407 ^b^	4.221 ^a,b^	**	NS	NS
C13:0	1.229	1.393	1.104 ^a^	1.370 ^a,b^	1.422 ^b^	1.349 ^a,b^	NS	NS	NS
C14:0	9.798	10.150	9.792 ^a^	9.958 ^a^	10.399 ^b^	9.747 ^a^	**	***	NS
C14:1	0.342	0.368	0.347	0.369	0.367	0.338	NS	NS	NS
C15:0	0.826	0.829	0.888 ^a^	0.875 ^a^	0.793 ^b^	0.755 ^b^	NS	***	NS
C15:1	0.237	0.251	0.248 ^a^	0.264 ^a^	0.222 ^b^	0.240 ^a^	NS	**	NS
C16:0	27.659	27.589	27.581	28.284 ^a^	27.639	26.993 ^b^	NS	**	NS
C16:1	0.666	0.663	0.607 ^a^	0.621 ^a^	0.761 ^b^	0.669 ^a^	NS	***	NS
C17:0	0.682	0.686	0.725 ^a^	0.715 ^a^	0.643 ^b^	0.653 ^b^	NS	***	NS
C17:1	0.240	0.263	0.228 ^a^	0.288 ^b^	0.248 ^a^	0.242 ^a^	*	**	NS
C18:0	12.852	12.079	12.976 ^a^	12.173 ^a,b^	11.853 ^b^	12.859 ^a^	*	NS	NS
C18:1 *trans*-11	0.557	0.567	0.607	0.556	0.492	0.592	NS	NS	NS
C18:1 *trans*-11 (VA)	1.327	2.116	1.285 ^a^	3.177 ^b^	1.154 ^a^	1.271 ^a^	*	***	***
C18:1 *cis*-9	22.173	21.293	21.812 ^a^	19.967 ^b^	22.420 ^a^	22.732 ^a^	NS	***	***
C18:2 *n*−6 *trans*	0.956	0.932	0.974 ^a,b^	0.790 ^a^	0.946 ^a,b^	1.066 ^b^	NS	**	NS
C18:2 *n*−6 *cis*	2.345	2.224	2.244 ^a^	1.858 ^b^	2.430 ^a,c^	2.606 ^c^	NS	***	NS
C18:3 *n*−6	0.292	0.338	0.301 ^a,b^	0.460 ^a^	0.243 ^b^	0.177 ^a,b^	NS	NS	NS
C18:3 *n*−3	0.580	0.526	0.664 ^a^	0.605 ^a,b^	0.487 ^b,c^	0.454 ^c^	NS	**	NS
C18:2 *cis*-9 *trans*-11 (CLA)	0.463	0.391	0.377	0.485	0.418	0.430	NS	NS	NS
C20:0	0.321	0.266	0.291	0.322	0.284	0.276	*	NS	NS
C20:5	0.123	0.122	0.102	0.141	0.111	0.126	NS	NS	NS
Δ^9^-desaturase activity									
C14:1/C14:0	0.035	0.036	0.035	0.037	0.035	0.035	NS	NS	NS
C16:1/C16:0	0.024	0.024	0.022 ^a^	0.022 ^a^	0.028 ^b^	0.025 ^a,b^	NS	***	NS
C18:1 *cis*-9/C18:0	1.764	1.794	1.710 ^a^	1.686 ^a^	1.929 ^b^	1.793 ^a,b^	NS	*	NS
CLA/VA	0.317	0.330	0.235 ^a^	0.417 ^b^	0.337 ^a,b^	0.304 ^a,b^	NS	*	NS

Superscripts a,b,c differ at *p* < 0.05; * = *p* < 0.05; ** = *p* < 0.01; *** = *p* < 0.001. NS = not significant; PS = production system; S = season.

**Table 5:** Effects of production system and season on milk lipid classes (% of total identified fatty acids) and *p*- values for the main effects and their interaction.

Lipid Class	Production Systems		Seasons				Main Effects		Interaction
	Conventional *n* = 94	Organic *n* = 66	Spring *n* = 41	Summer *n* = 40	Autumn *n* = 42	Winter *n* = 37	PS	S	PS × S
SCSFA ^1^	12.565	12.972	13.178 ^a^	12.825 ^a,b^	12.417 ^b^	13.178 ^a^	NS	NS	NS
MCSFA ^2^	43.601	44.332	43.574 ^a,b^	44.567 ^a^	44.660 ^a^	43.574 ^b^	NS	*	NS
LCSFA ^3^	13.783	12.962	13.905 ^a^	13.143 ^a,b^	12.765 ^b^	13.905 ^a^	*	NS	NS
SFA ^4^	69.949	70.266	70.657 ^a^	70.535 ^a^	69.841 ^a,b^	69.396 ^b^	NS	*	NS
MUFA ^5^	25.554	25.525	25.136 ^a^	25.254 ^a^	25.684 ^a,b^	25.136 ^b^	NS	NS	NS
PUFA ^6^	4.497	4.209	4.207	4.211	4.475	4.207	NS	NS	NS
UFA ^7^	30.051	29.734	29.343 ^a^	29.465 ^a^	30.159 ^a,b^	30.604 ^b^	NS	*	NS
OCFA ^8^	3.409	3.618	3.383 ^a^	3.719 ^b^	3.526 ^a,b^	3.427 ^a,b^	NS	NS	NS
*n*−3	0.581	0.505	0.583	0.628	0.523	0.438	NS	NS	NS
*n*−6	3.500	3.344	3.324 ^a^	3.108 ^a^	3.566 ^a,b^	3.691 ^b^	NS	**	NS

^1^ = short-chain saturated fatty acids; ^2^ = medium-chain saturated fatty acids; ^3^ = long-chain saturated fatty acids; ^4^ = saturated fatty acids; ^5^ = monounsaturated fatty acids; ^6^ = polyunsaturated fatty acids; ^7^ = unsaturated fatty acids; ^8^ = odd-chain fatty acids. Superscripts a,b differ at *p* < 0.05; * = *p* < 0.05; ** = *p* < 0.01. NS = not significant; PS = production system; S = season.

**Table 6:** Effects of production system and season on milk fat nutritional indices and *p*- values for the main effects and their interaction.

Index	Production Systems		Seasons				Main Effects		Interaction
	Conventional *n* = 94	Organic *n* = 66	Spring *n* = 41	Summer *n* = 40	Autumn *n* = 42	Winter *n* = 37	PS	S	PS × S
AI ^1^	2.376	2.451	2.425 ^a,b^	2.466 ^a^	2.455 ^a^	2.309 ^b^	NS	NS	NS
TI ^2^	3.080	3.105	3.131	3.113	3.071	3.054	NS	NS	NS
h/H ^3^	0.646	0.609	0.628 ^a^	0.577 ^b^	0.637 ^a^	0.668 ^a^	*	***	***
HPI ^4^	0.426	0.411	0.415 ^a,b^	0.411 ^a^	0.412 ^a^	0.438 ^b^	NS	NS	NS
LA/ALA ^5^	4.989	5.297	4.161 ^a^	3.671 ^a^	5.789 ^b^	6.952 ^b^	NS	***	NS
TFA ^6^	1.513	1.499	1.582	1.346 ^a^	1.438	1.658 b	NS	NS	NS
DFA ^7^	42.903	41.813	42.319	41.638 ^a^	42.011	43.463 ^b^	*	NS	NS
PUFA/SFA ^8^	0.065	0.060	0.060	0.060	0.064	0.065	NS	NS	NS

^1^ = atherogenicity index; ^2^ = thrombogenicity index; ^3^ = hypocholesterolemic: hypercholesterolemic ratio; ^4^ = health-promoting index; ^5^ = linoleic acid/α-linolenic acid ratio; ^6^ = trans fatty acids; ^7^ = desirable fatty acids; ^8^ = polyunsaturated fatty acid/saturated fatty acid ratio. Superscripts a,b differ at *p* < 0.05; * = *p* < 0.05; *** = *p* < 0.001. NS = not significant; PS = production system; S = season.

## Data Availability

The data presented in this study are contained within the article.

## Supplementary Materials

The following supporting information can be downloaded at: https://www.mdpi.com/article/10.3390/ani12172204/s1, Table S1: Milk chemical composition (g/100 g) of retail goat milk purchased in Greece in 2019–2020 for 12 months, Figures S1–S3: Principal Component Analysis for individual fatty acids, production system and season.

## Abbreviations

AI	Atherogenicity index
CLA	Conjugated fatty acid
DFA	Desirable fatty acid
EFSA	European Food Safety Authority
h/H	Hypocholesterolemic/hypercholesterolemic ratio
HPI	Health-promoting index
LA/ALA	Linoleic acid/α-linolenic acid ratio
LCSFA	Long-chain saturated fatty acid
MCSFA	Medium-chain saturated fatty acid
MCT	Medium-chain triglyceride
MUFA	Monounsaturated fatty acid
OCFA	Odd-chain fatty acid
PUFA	Polyunsaturated fatty acid
SCSFA	Short-chain saturated fatty acid
SFA	Saturated fatty acid
TFA	Trans fatty acid
TI	Thrombogenicity index
VA	Vaccenic acid
