# Supplementary material for: Effects of Production Systems and Seasons on Retail-Goat-Milk Fatty-Acid Composition and Nutritional Indices in Greece
Source: Animals (Basel). 2022 Aug 26;12(17):2204. doi: 10.3390/ani12172204 (PMC9454797; doi:10.3390/ani12172204)
Supplement: Supplementary file 1 [file animals-12-02204-s001.zip › animals-1859350-supplementary.pdf]

Table S1. Chemical composition (g/100 g) of retail goat milk purchased in Greece during 2019–2020 for 12 months

Brand No	Production System	Fat	SFA [1]	Protein	Carbohydrates	Sugars
1	Organic	3.24	2.04	3.24	4.26	4.17
2	Conventional	3.24	2.22	3.33	4.26	4.17
3	Organic	3.80	2.96	3.52	3.80	3.80
4	Conventional	3.80	2.96	3.52	3.80	3.80
5	Organic	2.59	2.04	3.52	4.07	4.07
6	Conventional	2.59	2.04	3.52	4.07	4.07
7	Conventional [2]	3.50	2.20	3.80	4.50	4.50
8	Conventional	3.70	2.69	3.43	3.98	3.98
9	Conventional	3.24	2.13	3.43	4.17	4.17
10	Conventional	3.43	2.41	3.33	4.17	4.17
11	Conventional	3.24	1.99	4.07	4.07	3.33
12	Conventional	3.24	2.04	4.17	4.17	3.33
13	Conventional	3.24	2.04	4.17	4.17	3.33
14	Organic [2]	3.50	2.20	3.80	4.10	4.10
Average (all samples)		3.27	2.26	3.61	4.07	3.86
Average (conventional)		3.37	2.25	3.65	4.10	3.85
Average (organic)		3.43	2.27	3.52	3.98	3.89

[1] SFA Saturated Fatty acids.

[2] Compositional data was available in g/100g

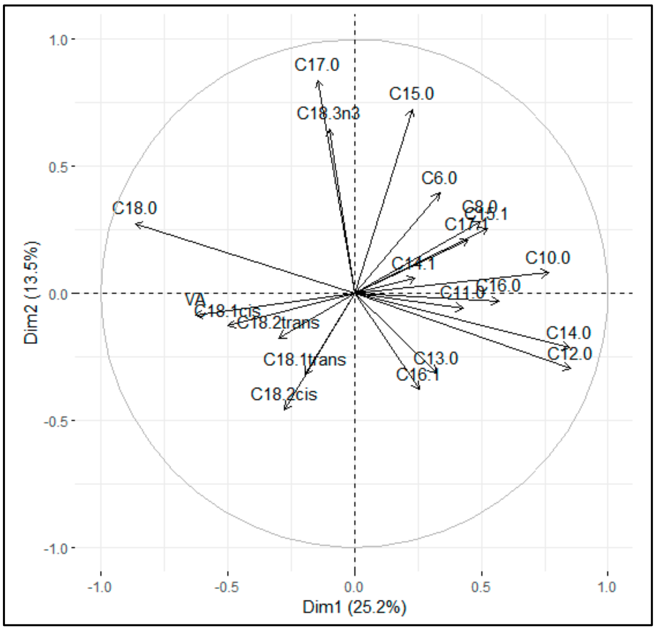

Figure S1. Principal Component Analysis for individual fatty acids. Positively correlated variables are grouped together. Negatively correlated variables are positioned on opposite sides of the plot origin (opposed quadrants). The distance between variables and the origin measures the quality of the variables on the factor map.

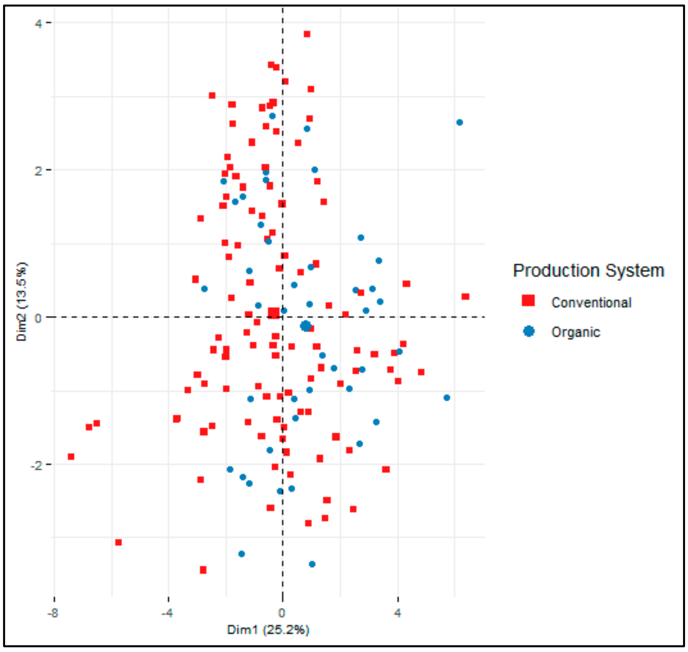

Figure S2. Principal Component Analysis - Components’ values grouped by Production System.

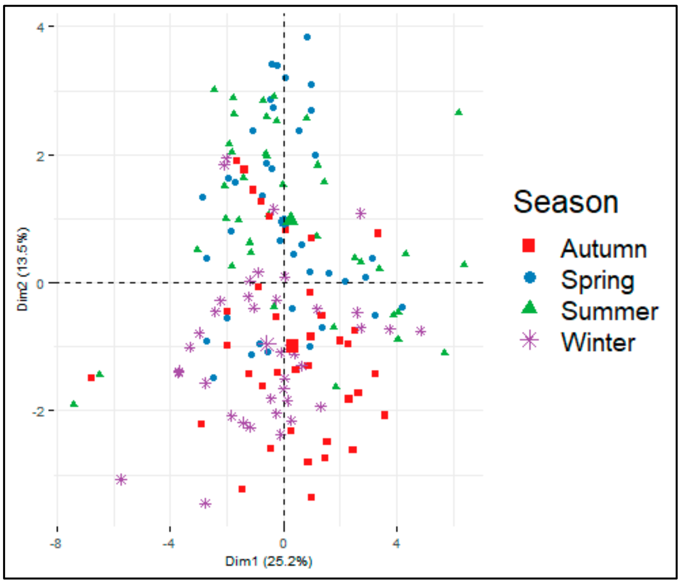

Figure S3. Principal Component Analysis - Components’ values grouped by Season.
